# Lower trapezius transfer to infraspinatus in cases of sequelae of obstetric brachial plexus injury

**DOI:** 10.1186/s12891-024-08048-y

**Published:** 2024-11-18

**Authors:** Mohammed A. Hasan, Amr El-Sayed, Mostafa Ezzat, Yasser Safoury

**Affiliations:** 1 Orthopedic and Trauma Surgery, New Valley University, ELkharga City, Egypt; 2 Head of the microsurgery Unit, Assiut University, Assiut City, Egypt; 3 Orthopedic and Trauma Surgery, Cairo University, Cairo City, Egypt

**Keywords:** Tendon transfer, Obstetric brachial plexus, Lower trapezius

## Abstract

**Background:**

Deficient shoulder function is a common and exhausting issue in children with obstetric brachial plexus injuries. Even with functioning elbow, wrist, and fingers, upper limb function is markedly disabled by limited shoulder abduction external rotation. Lower trapezius transfer carries many advantages; simple and safe technique, same line of pull as donor; reliable nerve supply (extraplexal from spinal accessory nerve), and not acting on rotation of the shoulder, mostly it will not adversely affect internal rotation range after the transfer. This study aims to evaluate the role of isolated lower trapezius transfer in reconstructing shoulder external rotation.

**Materials and methods:**

This prospective case series study included 20 patients with sequelae of obstetric brachial plexus injury lacking shoulder external rotation who underwent lower trapezius transfer to infraspinatus. In all cases, the lower trapezius muscle was the donor, and the recipient tendon was the Infraspinatus muscle. Shoulder range of motion, Modified Gilbert grading, and Mallet Classification were used to evaluate results.

**Results:**

The mean age at the time of surgery was 4.5 years. The average increase in shoulder external rotation and abduction was 40⁰ and 42.5⁰ respectively, the modified Gilbert grading improved from a mean of 3.85 to 4.85 postoperative. Mallet classification improved from a mean of 3.5 preoperative to 4.8 postoperative. Improvement (Mallet classification of ≥ 4) was obtained in 18 cases (90%).

**Conclusion:**

Isolated lower trapezius transfer is considered an effective option with promising results in cases of sequelae of obstetric brachial plexus injury for restoration of shoulder external rotation as well as abduction.

## Introduction

A tendon transfer is considered a surgical option in case of insufficient spontaneous recovery or residual functional impairements after microsurgical brachial plexus exploration [1, 2]. Choices available are debatable given the fact that the child is already in a deficit; lack of dispensable donors [3–6]. Persistent loss of shoulder abduction external rotation is seen in infants with different types of obstetric brachial plexus injuries [4, 7]. The possible presence of multiple denervated muscle groups and the persistent difficulty associated with the evaluation of clinical and functional deficits in such young children make them a challenging patient population [8–10].

Although previous studies about the subject of tendon transfers to restore shoulder abduction-external rotation do exist, this study is concerned with isolated lower trapezius transfer as the single donor (only lower trapezius transfer) in primary tendon transfer to restore external rotation deficit in OBPI cases.

## Materials and methods

A prospective case series study was performed on twenty patients who underwent lower trapezius transfer for improvement of shoulder function in two level one-microsurgery units, between 2021 and 2023. Indications of the surgery include children with OBPI with residual deficient shoulder function mainly external rotation so the child suffers with approaching the external environment as eating and overhead activities.Hand and elbow function should be well otherwise, different surgical planning should be carried out (this is not the case in our series). Also shoulder joint itself should be supple as a basic principle for any tendon transfer. Young age is required so the brain can adopt changes in the orientation of shoulder after the transfer.

Preoperative evaluation included Patient characteristics, such as age, sex, type of birth palsy, associated procedures, the diagnosis of OBPP was made based on traumatic delivery as well as clinical findings of injury (including limited active shoulder external rotation). X rays were done to all cases preoperative to exclude shoulder dysplasia. All cases were done by two senior hand surgeons (Y.S. and A.E). The donor tendon was the lower trapezius muscle to the infraspinatus muscle in all cases.

Postoperative Evaluation Tools included Shoulder range of motion; active and passive, Modified Gilbert Grading, and Modified Mallet Classification.

### Surgical technique

**Fig. 1:**
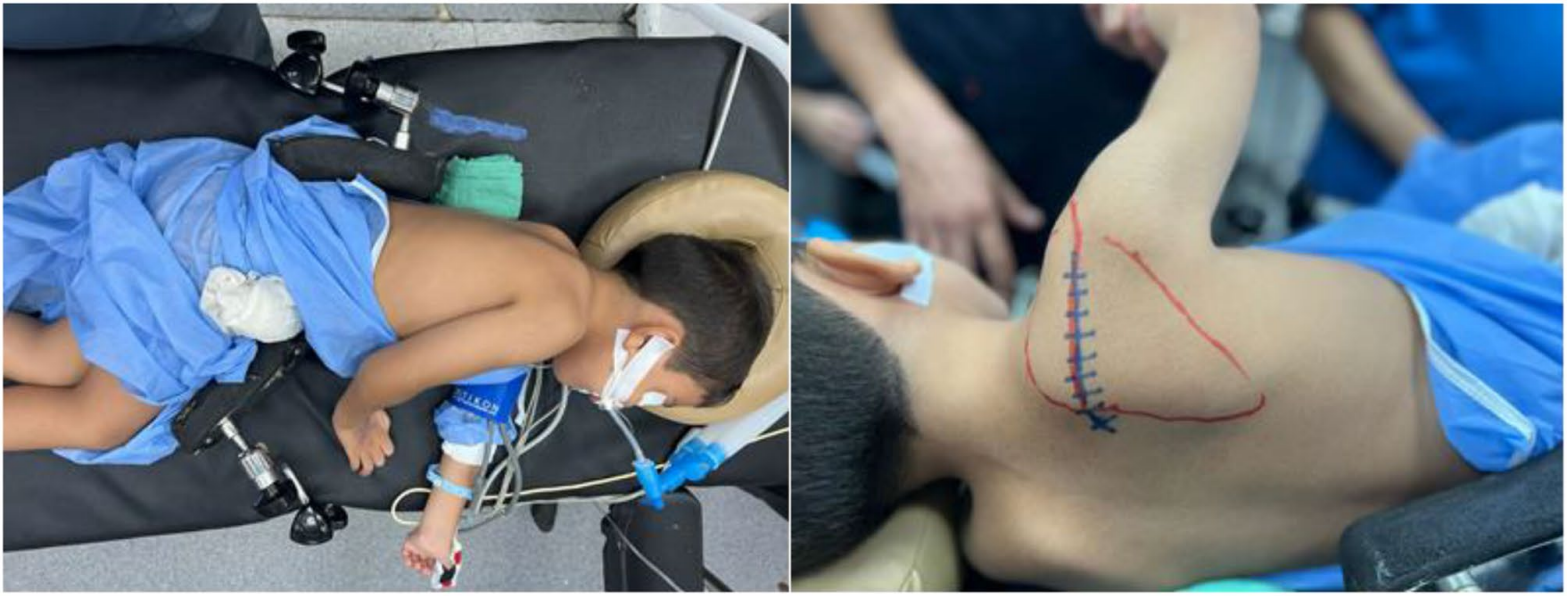
Patient positioning and marking of incision

**Fig. 2:**
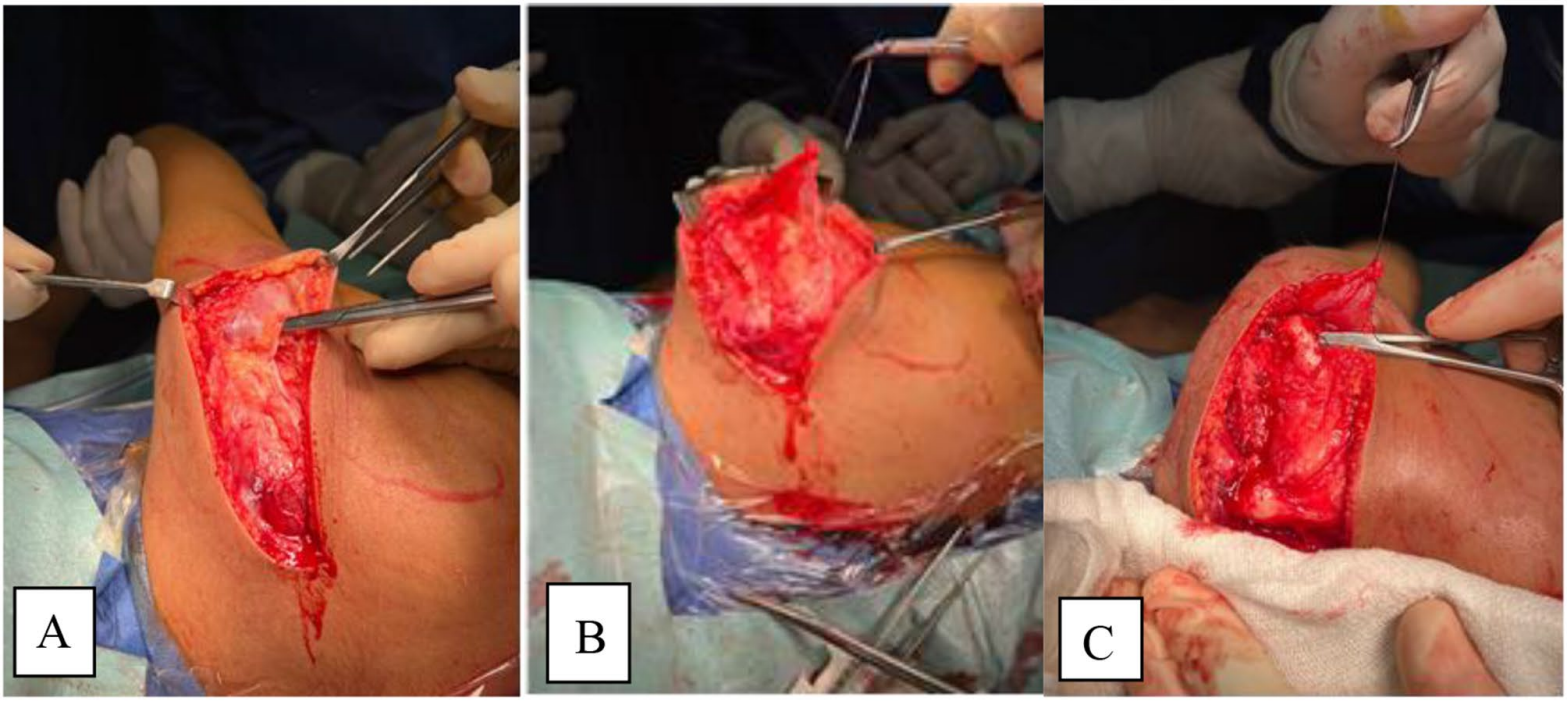
Identification of the recipient. **A** Posterior deltoid is attached to the lateral aspect of the scapular spine. **B** Posterior deltoid after elevation marked by stitches. **C** Infraspinatus tendon deep to posterior deltoid

**Fig. 3:**
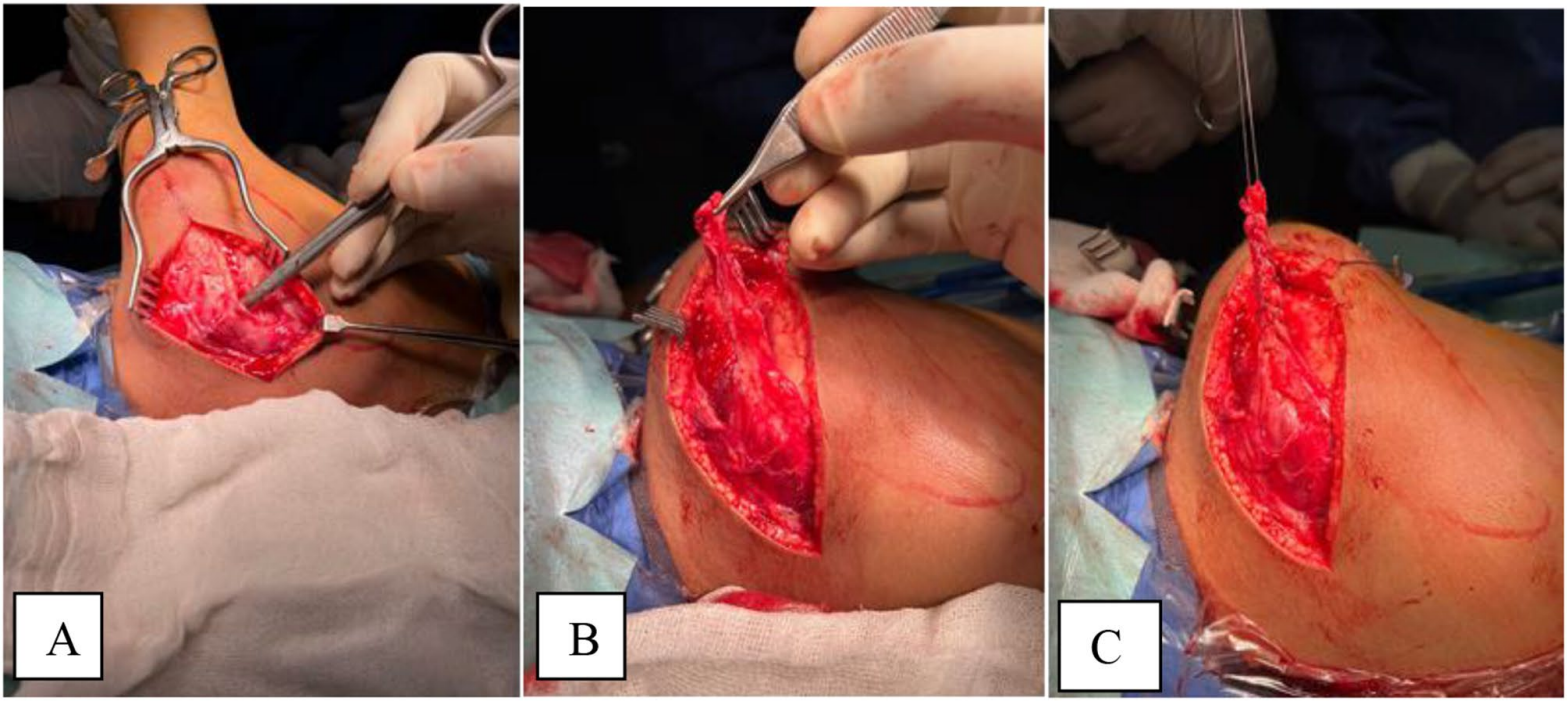
Identification of the donor. **A** Lower trapezius attached to the spine of the scapula. **B** Separation of its fibers with 2 cm periosteal extension. **C** Holding by stitches

**Fig. 4:**
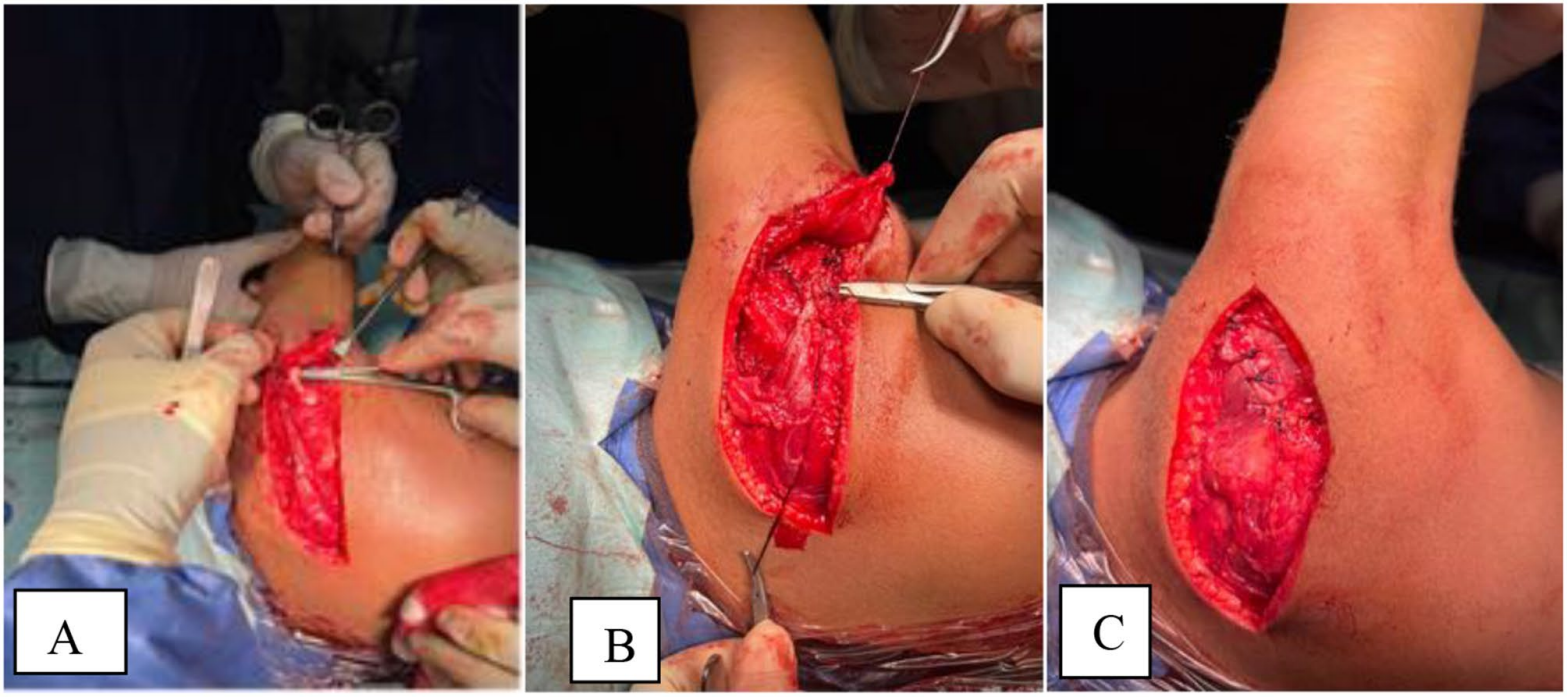
Completion of the transfer. **A&B** Suturing donor to recipient **C** Reattachment of posterior deltoid

The procedure is done according to Valenti et al. [11]. Under General anesthesia, the patient is positioned in a lateral position with the affected side uppermost. A straight incision was made along the spine of the scapula (Fig. 1), Identification of the donor and detachment of the lower fibers of the trapezius from the inferior border of the spine of scapula (Fig. 2), Identification of deltoid muscle origin on the lateral aspect of the scapular spine, then partial detachment of deltoid fibers from the lateral aspect of scapular spine to expose the underlying the tendon of infraspinatus (Fig. 3), Suturing of the tendinous aspect of lower trapezius tendon to the infraspinatus tendon with tension with the shoulder in maximal external rotation, Reattachment of deltoid fibers to bone (Fig. 4), Closure of the wound in layers and Shoulder immobilized in shoulder spica in maximal external rotation and 70 degrees abduction.

#### Postoperative

**Fig. 5:**
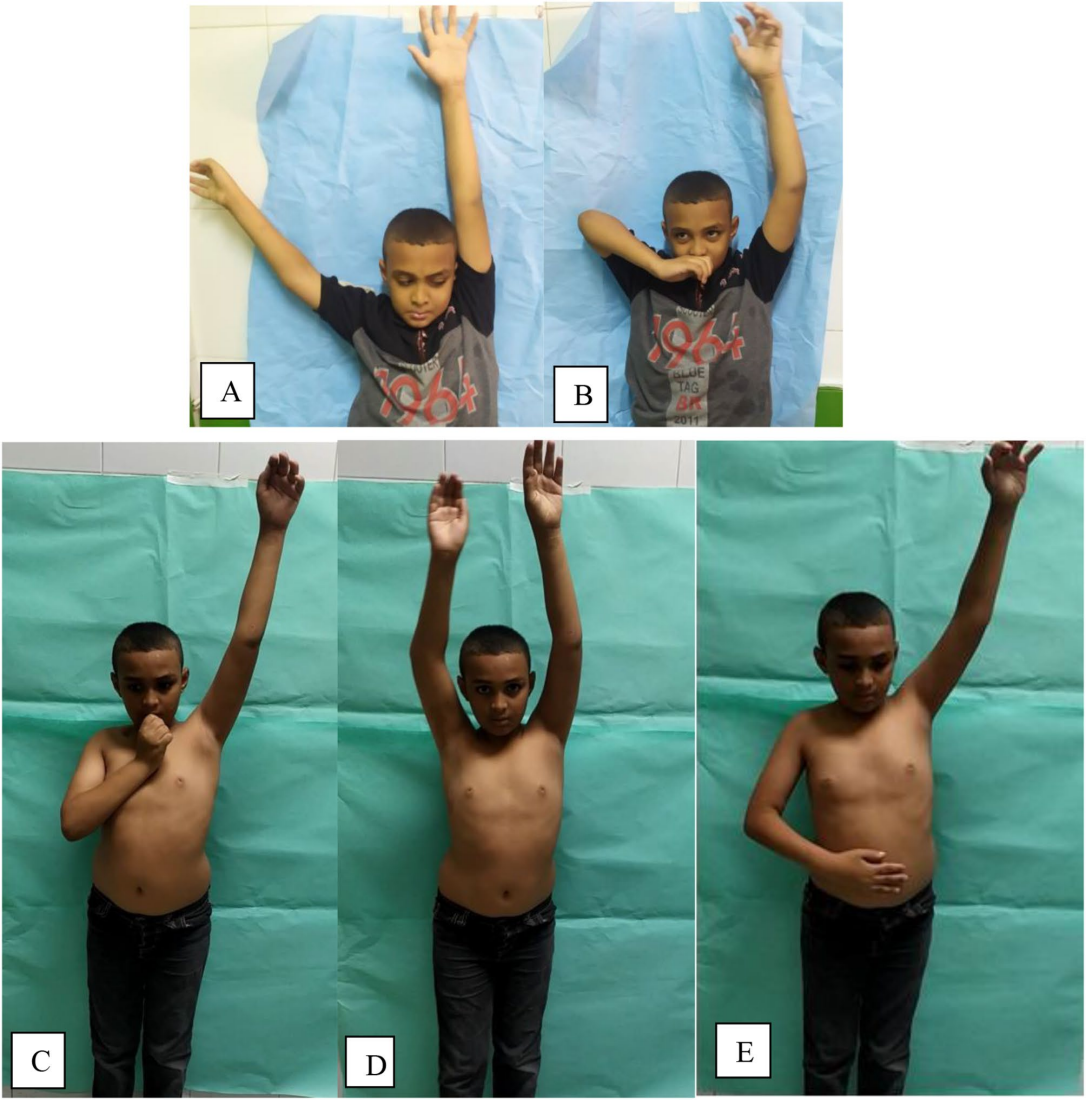
Male patient 9 years with Rt side OBPI. **A** Preoperative abduction. **B** Positive Trumpet sign. Postoperative (6 months) **C** abduction. **D** Postoperative hand to mouth. **E** Maintained good internal rotation

**Fig. 6:**
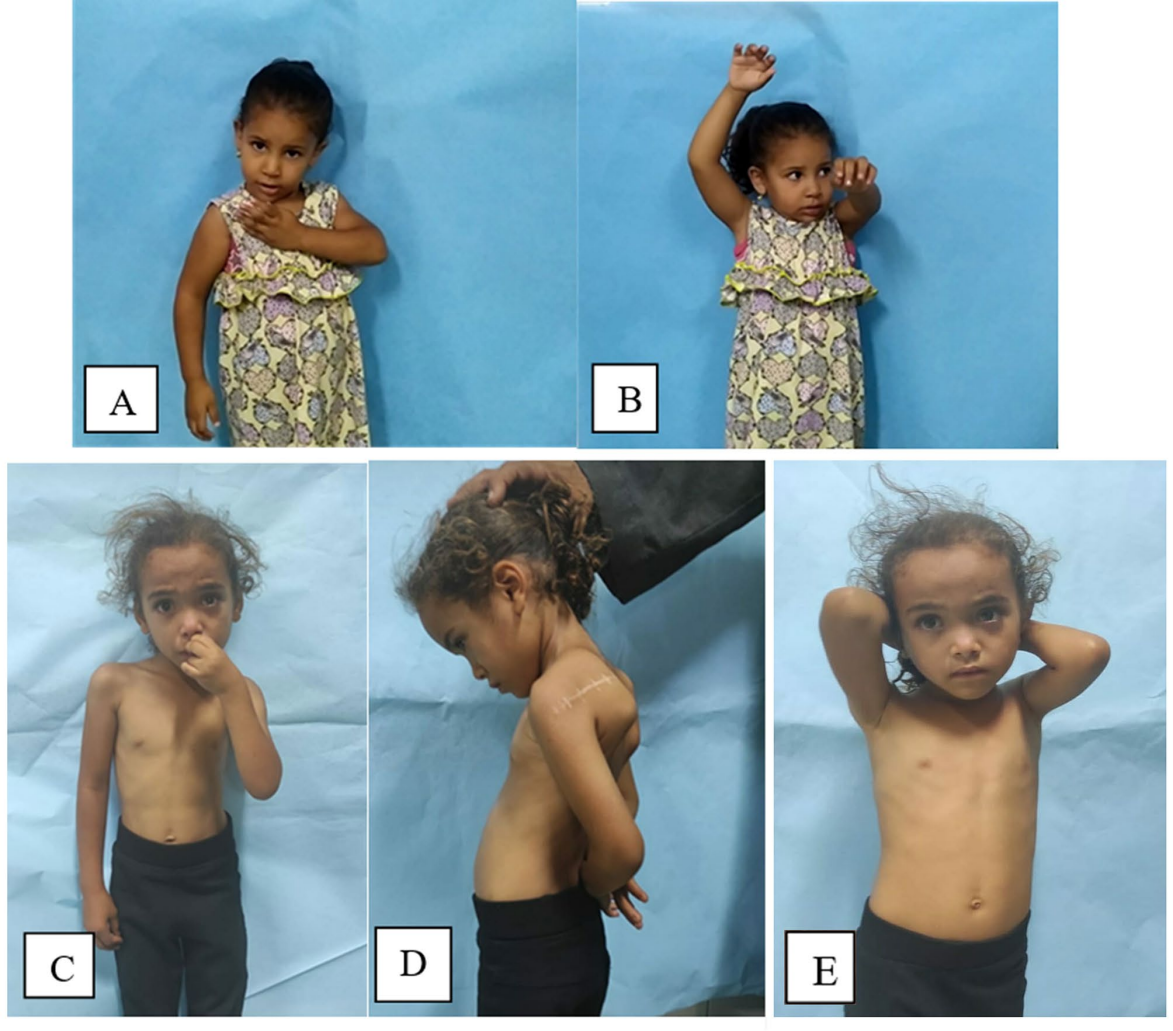
Female patient 4 years with Lt side OBPI. **A** Preoperative hand-to-mouth. **B** Preoperative abduction. Postoperative (6 months) **C** hand to mouth. **D** Maintained good internal rotation. **E** Postoperative external rotation

- Rehabilitation
  - Shoulder spica for 6 weeks then start active and passive range of motion exercises all patients followed the same postoperative protocol [12].
- Follow-up regimen
  - 6th week for spica removal and beginning of shoulder motion and.
  - 3rd, 6^th,^ and 12th months for the impact of the procedure on shoulder function (Figs. 5 and 6).

## Results

The age ranged from 2.5 to 9 years with a mean of 4.5 years. Seven patients were males and thirteen were females. Fourteen patients underwent tendon transfer to their Rt shoulder and six to their LT shoulder. The average gain in shoulder external rotation and abduction was 40⁰ and 42.5⁰ respectively, The modified Gilbert grading improved from a mean of 3.85 to 4.85 postoperative. Mallet classification improved from a mean of 3.5 preoperative to 4.8 postoperative. Two cases of wound infection (10%) were managed by antibiotics and improved. Nine patients (45%) developed keloids or hypertrophic scar. No cases of deteriorated internal rotation (external rotation contracture) were found. Young age (less than 4 years) was associated with slightly better results although they were not statistically significant.

Results on average are satisfactory in 90% of cases; Mallet classification ≥ 4 (Table 1).

**Table 1:** Results

	Range	Mean ± SD
**Age**	2–9	4.75 ± 2.56
**Operation Time**	40–90	64.5 ± 11.91
**Shoulder Ex Rotation**		
Pre	10–40	15.5 ± 8.26
Post	10–80	53 ± 21.3
**Shoulder Abd.**		
Pre	80–130	99 ± 10.21
Post	90–180	146 ± 22.28
**Modified Gilbert Grading**		
Pre	3–4	3.85 ± 0.37
Post	3–6	4.85 ± 0.67
**Mallet classification**		
Pre	3–4	3.5 ± 0.51
Post	3–5	4.1 ± 0.52

Table 2 showing the results using Wilcoxon test.

**Table 2:** Comparison between preoperative and postoperative results among patients using Wilcoxon test

	preoperative	postoperative	*P*. value
Median (IQ)			
ER	10(10–40)	50(10–80)	< 0.001**
Abd.	100(80–130)	146(90–180)	< 0.001**
Modified Gilbert Grading	3.8(3–4)	5(3–6)	< 0.001**
Mallet classification	14.5(13–16)	20(15–25)	< 0.001**

## Discussion

Lower trapezius transfer carries many advantages; Simple technique, same line of pull as receiver, reliable nerve supply (extraplexal from spinal accessory nerve), and being a muscle not acting on rotation of the shoulder, mostly it will not adversely affect internal rotation range after the transfer. The approach is safe not exposing important neurovascular structures apart from spinal accessory nerve [13, 14].

To our knowledge previous publications about lower trapezius transfer discussed it in the scene of revision cases, traumatic cases, adults cases with rotator cuff reconstruction or combined with other procedures. This series present the lower trapezius in fresh cases as a single operative procedure in children with OBPI and showed improvement in shoulder external rotation and abduction as well as quality of life. So evaluation of reports in the literature on trapezius transfer may be difficult due to the variations in the indications used in each study, and a comparison of the different outcomes may be inappropriate. In the case of previous microsurgical brachial plexus exploration, 2 years should pass to assure that no further recovery would occur so tendon transfer can be done if the recovery is not satisfactory [10, 15, 16].

Anterior shoulder soft tissue release should be considered if a difference in the passive range of external rotation between the affected and the normal side by ≥ 30⁰ exists [17, 18]. Healthy shoulder joints are obligatory to perform tendon transfer otherwise bony procedures should be carried out [2, 14, 19].

**Terzis et al.** underwent palliative surgery in 67 patients (68 extremities) for improving shoulder function.

These patients received different soft-tissue releases and/or tendon transfers, pedicled and/or free.

muscle transfers, and humeral osteotomies. A total of 197 secondary procedures were performed with a mean follow-up of 7.5 years (range, 12 months to 22 years).

They concluded that The concurrent transfer of the trapezius with other pedicle muscles, such as the.

latissimus dorsi or pectoralis major, has given significantly better results than the transfer of the trapezius alone and/or free muscle transfers for the management of shoulder sequelae in OBPI [10].

**Bertelli** et al. stated their results on seven patients, with a mean age of seven years (4 to 9 years). All had Erbs palsy and all of them were recurrent cases. Two years postoperatively, the mean increase in active and passive external rotation was 47.1° (20° to 70°), increasing to 54.3° (50° to 75°) at four years. These values described as statistically significant (*p* = 0.0004) [20]. 

**CHEN** et al. reported upon 34 children with limitation of the shoulder abduction and external rotation, as well as co-contraction of the shoulder adductors in abduction. They underwent transfer of the latissimus dorsi tendon with attached teres major to the infraspinatus (single procedure) in 25 patients, and transfer of both latissimus dorsi with teres major and trapezius (to the humerus) in nine cases (combined procedure). They declared that shoulder Abduction was improved in only 13 of the 25 cases with a single procedure and eight of 9 cases with a combined transfer. They concluded that for improving both abduction and external rotation of the shoulder in OBPP, transfer of the latissimus dorsi (along with teres major) can be performed only when shoulder abduction is 90° or more; otherwise, lower trapezius transfer should be added [21].

**T.H. Abdelaziz et al.** reported on 35 children with upper trunk OBPI palsy and shoulder abduction of < 90° underwent transfer of teres major. In 18 cases (group 1), a trapezius transfer was added (combined transfer). In 17 cases (group 2) only teres major transfer was done (single transfer). The mean increase in shoulder abduction was 67.2° (60° to 80°) in group 1 and 37.6° (20° to 70°) in group 2, which reached statistical significance (*p* < 0.001) [22].

The current study discusses the lower trapezius as a single transfer with very promising short-term results.

Limitations of this study include the small sample size and relatively short period of follow-up.

Finally, it should be mentioned that in many patients with severe brachial plexus birth injuries, the role of a tendon transfer is often palliative. Achieving best shoulder function would involve additional carefully selected procedures according to ongoing recovery.

## Data Availability

The datasets used and/or analysed during the current study are available from the corresponding author on reasonable request.

## Declarations

### Concent for publication

Patients’ legal guardians signed informed consent to publish their data and photographs showing human faces.

## Abbreviations

OBPI: Obstetric brachial plexus injury
ER: External rotation
ABD: Abduction
